# Antiplasmodial Screening of Phikud Navakot Formulation and In Vivo Evaluation, Toxicity, and Phytochemical Profiling of the Potent *Terminalia chebula* Gall Aqueous Extract

**DOI:** 10.1155/sci5/9598524

**Published:** 2025-11-28

**Authors:** Arisara Phuwajaroanpong, Chuchard Punsawad, Prapaporn Chaniad, Atthaphon Konyanee, Abdi Wira Septama, Walaiporn Plirat

**Affiliations:** ^1^Department of Medical Technology, School of Allied Health Sciences, Walailak University, Nakhon Si Thammarat 80160, Thailand; ^2^Center of Excellence in Tropical Pathobiology, Walailak University, Nakhon Si Thammarat 80160, Thailand; ^3^Department of Medical Sciences, School of Medicine, Walailak University, Nakhon Si Thammarat 80160, Thailand; ^4^Research Center for Pharmaceutical Ingredient and Traditional Medicine, Cibinong Science Center, Cibinong Science Center, National Research and Innovation Agency (BRIN), Jakarta, West Java, Indonesia

## Abstract

The growing resistance to first-line artemisinin-based therapies underscores the urgent need for novel antimalarial agents, with medicinal plants offering a promising source of candidates. Phikud Navakot formulation and its component plants are widely used in traditional medicine; however, their antimalarial properties remain underexplored. This study aimed to screen the Phikud Navakot formulation and its individual component plants for antiplasmodial activity and to further evaluate the most potent extract through in vivo efficacy testing, acute toxicity assessment, and phytochemical profiling. In vitro antiplasmodial activity was evaluated against a chloroquine-resistant *Plasmodium falciparum* K1 strain, and in vivo efficacy was assessed using the standard 4-day suppressive test in *P. berghei* ANKA–infected mice. Acute oral toxicity was examined in mice at a limit dose of 2000 mg/kg. Among all crude extracts tested, the aqueous gall extract of *Terminalia chebula* exhibited potent in vitro antiplasmodial activity (IC_50_ = 3.24 ± 0.83 μg/mL) without toxicity (CC_50_ > 100 μg/mL) in Vero cells or hemolytic effects. In the 4-day suppressive test, mice treated with 200, 400, and 600 mg/kg doses of the aqueous *T. chebula* gall extract showed significant, dose-dependent suppression of parasitemia (38.88%, 47.08% and 60.61%, respectively; *p* < 0.05). Furthermore, no signs of acute toxicity were observed at the 2000 mg/kg dose. Phytochemical profiling indicated that the aqueous *T. chebula* gall extract contains multiple bioactive compounds with potential medicinal properties. Overall, these findings demonstrate that the aqueous *T. chebula* gall extract possesses promising both in vitro and in vivo antimalarial activities, with an excellent safety profile. This provides scientific evidence supporting its potential as a plant-based candidate for antimalarial drug development. Further studies are warranted to isolate the active constituents, elucidate their mechanisms of action, and conduct subacute toxicity studies to extend the safety evaluation beyond the current findings.

## 1. Introduction

According to a World Health Organization (WHO) report, in 2024, an estimated 263 million malaria cases were recorded worldwide, resulting in approximately 600,000 deaths annually, with the majority of fatalities occurring in sub-Saharan Africa [1]. Currently, drug resistance has led to increasing rates of treatment failure, complicating malaria control efforts and contributing to sustained morbidity and mortality each year [2]. Chloroquine (CQ) has historically been one of the most widely used antimalarial drugs because of its cost-effectiveness, safety, and efficacy. However, widespread resistance to CQ is driven by genetic mutations in the *Plasmodium falciparum* (*P. falciparum*) CQ resistance transporter and *P. falciparum* multidrug resistance Protein 1 genes [3, 4]. Artemisinin-based combination therapies (ACTs) have increasingly encountered the emergence of resistance in several countries. Artemisinin partial resistance was first identified in the Greater Mekong Subregion—notably in Cambodia, Thailand, Myanmar, Laos, and Vietnam [5]. More recently, artemisinin partial resistance has been confirmed in several eastern African countries, including Rwanda [6]. The spread of artemisinin-resistant strains threatens global malaria elimination. Reliance solely on agents acting through existing pathways may compromise long-term treatment efficacy. Therefore, the development of compounds with distinct molecular targets is essential to overcome resistance, ensure sustainable therapeutic outcomes, and strengthen malaria control efforts [7].

Medicinal plants are rich in biologically active phytochemicals, such as alkaloids, flavonoids, terpenoids, saponins, and polyphenols, which demonstrate diverse biological activities, including antibacterial, antioxidant, immunomodulatory, and antiplasmodial properties [8, 9]. Owing to their therapeutic potential, medicinal plants have become a major focus in the search for new treatments, particularly for infectious diseases such as malaria [10]. Therefore, ongoing research aims to identify new plant-derived compounds with strong antimalarial activity, improved safety profiles, and novel mechanisms of action to address drug-resistant *Plasmodium* strains.

The Phikud Navakot formulation is a traditional Thai polyherbal remedy officially recognized in Thailand's National List of Essential Medicines (NLEM). The Phikud Navakot formulation is commonly used to alleviate circulatory disorders, dizziness, fainting, and various fevers, including malaria-like symptoms [11]. From pharmacological effects, it protects erythrocytes from oxidative damage [12], reduces ROS/RNS in endothelial cells [13], enhances blood flow via muscarinic receptors [14], and suppress neuroinflammation [15]. It inhibits ERK1/2 in microglial activation and amyloid buildup, and ER stress in Alzheimer's models [16]. The Phikud Navakot formulation comprises an equal weight proportions (1:1 ratio) of the nine dried raw materials [17] including *Angelica dahurica* (*A. dahurica*), *Atractylodes lancea* (*A. lancea*), *Ligusticum sinense* (*L. sinense*), *A. sinensis* (*A. sinensis*), *Artemisia pallens* (*A. pallens*), *Saussurea costus* (*S. costus*), *Picrorhiza kurrooa* (*P. kurrooa*), *Terminalia chebula* (*T. chebula*), and *Nardostachys jatamansi* (*N. jatamansi*) [18]. The plant components of Phikud Navakot formulation display several pharmacological properties (Table 1). Although ethnobotanical records do not explicitly report the antimalarial use of the Phikud Navakot formulation or its components, their diverse bioactive compounds and known pharmacological activities suggest potential benefits. These extracts may exert direct antiparasitic effects while also alleviating disease severity through immunomodulatory mechanisms, the regulation of inflammatory responses in this highly inflammatory condition, and the reduction of oxidative damage to host cells and tissues. Therefore, this study set out to assess the antiplasmodial potential of the Phikud Navakot formulation and each of its constituent plants, with subsequent in vivo evaluation, acute toxicity testing, and the phytochemical analysis of the most promising extract.

## 2. Materials and Methods

### 2.1. Collection, Authentication, and Extraction of Phikud Navakot Formula and Its Component Plants

The Phikud Navakot formula and its constituent plants were purchased from the Thai Traditional Pharmacy, Nakhon Si Thammarat, Thailand, in October 2024 (Table 1). Plant specimens were authenticated by Assistant Professor Dr. Jiratthi Satthaphorn, a botanist in the School of Science at Walailak University in Thailand. A voucher herbarium specimen was placed at Walailak University's School of Medicine. The extraction process followed a previously established protocol [33, 34]. To eliminate residues, the plant specimens were washed with filtered water before being placed in a hot-air oven set to 60°C for 72 h. After drying, the plant material was chopped, and 60 g of each sample was prepared. Phikud Navakot formulation was prepared from nine medicinal plants, each contributing an equal proportion (1:1 ratio). Specifically, 6.67 g of each plant material was weighed and combined, resulting in a total weight of 60 g. The plants and Phikud Navakot formulation were subjected to two extraction methods: maceration and decoction. For maceration, 60 g of dried material was soaked in 600 mL 95% ethanol (Merck, Darmstadt, Germany) at room temperature for 72 h. For decoction, 60 g of dried material was boiled with 600 mL of distilled water at 90°C–100°C for 30 min. Both methods were repeated twice to maximize the extraction. The extracts were filtered through Whatman No. 1 filter paper (GE Healthcare, USA), and the ethanol extract was evaporated at 45°C in a rotary evaporator for 4–5 h until dryness (Heidolph Hei-VAP, Schwabach, Germany). Both ethanolic and aqueous extracts were freeze-dried using a Christ Gamma 2–16 LSC plus freeze-dryer (Osterode am Harz, Germany) for 48 h to obtain the dried extract and stored in refrigerated containers at 4°C for later analysis.

### 2.2. Calculation of Percent Yield From Plant Extracts

The percentage (%) yield indicates the actual crude extract from the initial plant powder. The % yield was computed as the ratio of actual to starting plant weights. The % yield was computed using the following equation [35]:

(1) $$\begin{array}{c} \%\text{ yield}=\frac{\text{weight of dried plant extract }\left(g\right)}{\text{initial dry weight of plant material }\left(g\right)}\times 100. \end{array}$$

### 2.3. In Vitro Maintenance and Propagation of *P. falciparum* K1 Strain

The CQ-resistant *P. falciparum* K1 strain was cultivated using the method initially described by Trager and Jensen, with minimal changes [36]. Parasite cultivation was carried out using a Roswell Park Memorial Institute (RPMI) 1640 medium (Gibco, USA) supplemented with additives to promote optimal growth and sterility. The culture medium included 2 mg/mL sodium bicarbonate (Sigma-Aldrich, USA) for pH buffering, 10 μg/mL hypoxanthine (Sigma-Aldrich, USA) for purine synthesis, and 4.8 mg/mL HEPES buffer (HiMedia, India) for pH stability under atmospheric conditions. 0.5% (w/v) AlbuMAX II, a lipid-rich bovine serum albumin (Gibco, New Zealand), was used as a protein and lipid source. The medium was supplemented with 2.5 μg/mL gentamicin (Sigma-Aldrich, India) to prevent bacterial contamination during cultivation. Giemsa-stained thin blood smears were used to detect parasitemia, which was then viewed under a light microscope (Olympus CX31; Tokyo, Japan) with a 100X oil immersion lens. The cultures were subcultured every 24–48 h by adjusting the hematocrit (HCT) levels and replacing the medium. For stage-specific synchronization, ring-stage parasites were isolated using a 5% d-sorbitol solution, which lyses mature trophozoites and schizonts, while preserving the ring stages. Synchronized cultures were washed, resuspended in a complete medium, and maintained for further experiments.

### 2.4. Quantitative Evaluation of Antiplasmodial Efficacy via Parasite Lactate Dehydrogenase (pLDH) Assay in *P. falciparum* Cultures

The pLDH assay, originally developed by Makler et al. and subsequently modified for enhanced sensitivity and reproducibility [29], was used to assess the antiplasmodium activity of the test substances by quantifying the pLDH enzyme produced by viable *P. falciparum* parasites. To prepare stock solutions, ethanolic and aqueous extracts of the Phikud Navakot formulation and its components were dissolved in dimethyl sulfoxide (DMSO) (Merck, Germany) and RPMI medium. These solutions were then serially diluted to concentrations ranging from 0.78 to 100 μg/mL. Artemisinin (ARS) (Sigma-Aldrich) was utilized as a positive control, DMSO was used as a negative control, and uninfected erythrocytes were used as a baseline reference. Cultures of *P. falciparum* K1 strain–infected erythrocytes, standardized to 2% parasitemia and 2% HCT, were plated in 96-well microplates (199 μL per well). Test extracts, positive controls, and vehicle controls were added in triplicate (1 μL per well per concentration). The plates were incubated at 37°C with 5% CO_2_ for a period of 72 h. After the incubation process, parasite-infected erythrocytes underwent three freeze–thaw cycles (−20°C for 30 min, followed by 37°C for 30 min) to lyse the cells and release pLDH enzymes. To detect pLDH activity, 20 μL of each parasite lysate was transferred to a new 96-well microplate, followed by the addition of 100 μL of Malstat reagent. Subsequently, 20 μL of a chromogenic detection solution—comprising p-nitroblue tetrazolium chloride (NBT; Merck, Germany) and phenazine ethosulfate (PES; Sigma-Aldrich, USA)—was added to each well. The NBT/PES allows for the colorimetric detection of pLDH activity by producing a purple formazan product. The plates were subsequently left in the dark at room temperature for 1 h. Parasite viability was determined by measuring absorbance at 650 nm with a Multiskan SkyHigh microplate spectrophotometer (Thermo Fisher Scientific, USA).

### 2.5. Cultivation and Maintenance of Vero Cell Lines for Experimental Applications

Vero cells (ATCC Catalog No.: CCL-81, RRID: CVCL_0059), originally established in 1962 from kidney tissue of a normal adult African green monkey (*Chlorocebus sabaeus*), were cultured under standard conditions using Dulbecco's modified Eagle's medium (DMEM; Gibco, USA). The medium was supplemented with 10% heat-inactivated fetal bovine serum (FBS; Sigma-Aldrich, India) to supply essential nutrients and growth factors necessary for optimal cell proliferation. Additionally, 1% penicillin–streptomycin solution was included to prevent bacterial contamination and maintain aseptic conditions throughout the cultivation process. The cultures were maintained at 37°C in a humidified environment with 5% CO_2_ to support cell growth [37]. Cell confluence and morphology were monitored regularly using a phase-contrast inverted microscope (Olympus, Model CKX31, USA). Upon reaching 80% confluence, the cells were detached using 2.5% trypsin-EDTA (Gibco, USA), neutralized with complete medium, and subcultured at an appropriate dilution ratio to maintain growth.

### 2.6. In Vitro Cytotoxicity Assessment Using the 3-(4,5-Dimethylthiazol-2-yl)-2,5-Diphenyltetrazolium Bromide (MTT) Assay

The in vitro cytotoxicity of the test sample was assessed using the MTT assay following a well-established protocol with minor modifications [38]. Vero cells were seeded into 96-well microplates at 2 × 10^4^ cells per well in 199 μL of complete medium and allowed to adhere overnight. After attachment, 1 μL of each extract derived from the Phikud Navakot formulation and its components extract was added to achieve final concentrations of 1.53 to 100 μg/mL, with all treatments performed in triplicate. Doxorubicin (DX; Sigma-Aldrich, India) served as a positive control, and DMSO was used as a negative control. After 48 h, the culture medium was aspirated, and 50 μL of MTT solution (5 mg/mL in phosphate-buffered saline [PBS]; Thermo Fisher Scientific, USA) was added to each well. Plates were incubated for an additional 2 h. The supernatant was removed, and 100 μL of DMSO was added to dissolve the formazan crystals. The absorbance at 560 nm was measured with a Multiskan SkyHigh spectrophotometer (Thermo Fisher Scientific, USA), and the background absorbance at 670 nm was removed. The % cytotoxicity was determined using the following formula [39]:

(2) $$\begin{array}{c} \%\text{ cytotoxicity}=100-\left[100\times \frac{\text{optical density of sample well}}{\text{optical density of negative well}}\right]. \end{array}$$

### 2.7. Analysis of Selectivity Index (SI)

The SI was determined by comparing the CC_50_ value against Vero cells to the IC_50_ value against *P. falciparum* K1 strain, as given in the following equation [40, 41]:

(3) $$\begin{array}{c} \text{selectivity index}=\frac{{\text{CC}}_{50}\;\left(\text{Vero cells}\right)}{{\text{IC}}_{50}\;\left(P.\;falciparum\;K1\right)}. \end{array}$$

A SI value greater than 10 is generally considered indicative of a favorable therapeutic window, reflecting a significant margin between the antimalarial efficacy of the compound and its cytotoxicity to mammalian cells. Therefore, crude extracts with SI values exceeding this threshold and potent in vitro antimalarial activity were prioritized for in vivo efficacy and safety assessments.

### 2.8. Hemolysis Assay

The potential cytotoxicity of the crude ethanolic and aqueous extracts on human erythrocytes was assessed by measuring hemolysis. The identities of study participants were protected by assigning unique codes, removing all personal identifiers, and restricting data access to authorized personnel only. The study protocol was reviewed and approved by the Ethics Committee in Human Research Walailak University (Approval No. WUEC-24-350-01). Written informed consent was obtained from all blood donors prior to sample collection, and all procedures were conducted in accordance with the Declaration of Helsinki. Venous blood samples were collected from healthy adult volunteers under sterile conditions and placed in tubes containing ethylenediaminetetraacetic acid (EDTA) as an anticoagulant. The red blood cells (RBCs) were separated by centrifugation at 3000 rpm for 5 min, and the plasma and buffy coat layers were carefully discarded. The erythrocyte pellet was washed three times with sterile PBS to remove plasma proteins and residual leukocytes. A 2% HCT RBC suspension was prepared and incubated with 50 μg/mL of each plant crude extract in a final reaction volume of 0.2 mL in sterile microplates. The incubation was carried out at 37°C for 72 h in a humidified environment. Triton X-100 (Sigma-Aldrich, New Delhi, India) was used as a positive control to induce complete hemolysis. DMSO and PBS served as negative controls to assess baseline hemolysis levels. Following incubation, the microplates were centrifuged at 3000 rpm for 5 min to pellet intact cells. The supernatant from each well was transferred to a new 96-well microplate, and the absorbance corresponding to free hemoglobin release was measured at 570 nm using a microplate spectrophotometer. Extracts causing less than 10% hemolysis were classified as nonhemolytic, whereas those exceeding 25% were regarded as having a significant hemolytic effect on healthy human erythrocytes. The percentage of hemolysis was calculated using the following formula [42]:

(4) $$\begin{array}{c} \%\text{ hemolysis}=\frac{\left(\text{optical density of sample}-\text{optical density of negative control}\right)}{\left(\text{optical density of positive control}-\text{optical density of negative control}\right)}\times 100. \end{array}$$

### 2.9. Selection of the Extracts

The plant extracts and formulations were selected to identify potential candidates for further investigation. These candidates were evaluated through a series of assessments including in vitro antimalarial activity, and in vitro toxicity studies. The key criteria for selecting promising candidates were SI and hemolysis values. Therefore, the final selection of candidate extracts was based on those that demonstrated the highest SI values (greater than 10) and acceptable hemolysis values (less than 25%), ensuring both efficacy and safety for further experiment.

### 2.10. Application of Liquid Chromatography-Mass Spectrometry (LC-MS) in the Identification of Phytochemicals From Phikud Navakot Formula and Its Components

The selected plant extract was examined for the phytochemical compounds by LC-MS. The LC-MS analysis was carried out on a LC-QTOF-MS instrument, 1290 Infinity II LC-6545 Quadrupole-TOF (Agilent Technologies, USA) with an electrospray ionization (ESI) interface. The MS conditions involved an ESI probe in the negative mode with a scanning range of 100−1200 m/z. A Zorbax Eclipse Plus C18 column (100 mm length × 2.1 mm inner diameter, particle size 1.8 μm) (Agilent Technologies, USA) was used for chromatographic separation. The mobile phase contained a binary solvent system: (A) 2% acetic acid in water and (B) acetonitrile (CH_3_CN). The column temperature was set to 25°C. A flow rate of the mobile phase was 0.20 mL/min, and the injection volume was 2 μL. For tandem mass spectrometry (MS/MS) analysis, the instrument was programmed to automatically select and fragment the most abundant precursor ions in the m/z range of 100–1200, allowing for further structural elucidation of the compounds. The similarity score was used to identify compounds by comparing the retention durations and mass data of an unknown compound to reference spectra obtained from the METLIN mass spectra library (Agilent Technologies, USA). Compounds discovered in the extracts were identified using a similarity score greater than 80%.

### 2.11. Animal Preparation for 4-Day Suppressive Test

Healthy male Institute for Cancer Research (ICR) mice (also known as CD-1 mice; RRID: IMSR_CRL:022), a strain of albino *Mus musculus* originally developed from the Swiss albino lineage, were used in this study. The animals, aged 6–8 weeks and weighing between 20 and 30 g, were obtained from Nomura Siam International Co., Ltd., located in Pathumwan, Bangkok, Thailand. Prior to commencing the experiments, the animal research protocol was reviewed and approved by the Animal Ethics Committee of Walailak University, Thailand, to ensure adherence to ethical guidelines. The mice were housed under controlled environmental conditions, including a 12-h light/dark cycle, with temperature maintained at 22 ± 3°C and relative humidity set between 50% and 60%. They were provided a standard commercial pellet diet and access to water ad libitum. All mice were acclimatized for 1 week in a laboratory environment to minimize stress before the experiment began. The animals were randomly assigned to experimental groups, and all procedures involving animal handling and experimentation complied with the international guidelines for the ethical treatment and use of laboratory animals.

### 2.12. Experimental Grouping and Dose Administration in 4-Day Suppressive Test

To evaluate the in vivo antimalarial activity of selected crude extracts from the Phikud Navakot formulation, male ICR mice were infected with *P. berghei* and randomly assigned to six groups, with five mice per group (Table 2). Each group received a different crude extract at doses of 200, 400, or 600 mg/kg once daily for four consecutive days. The control groups included a negative control group, which was administered a solvent used for reconstitution (7% Tween 80), and a positive control group, which received the standard antimalarial drugs artesunate (ARS) (6 mg/kg) and CQ phosphate (25 mg/kg). These doses were selected based on previous research and the established efficacy of these compounds in the treatment of malaria [34].

### 2.13. Evaluation of Antimalarial Activity Using the 4-Day Suppressive Test (Peters' Test) in Mice Models

The antimalarial activity was evaluated using Peters' 4-day suppressive test [43]. The rodent malaria parasite, *P. berghei* ANKA strain, was provided by Thomas F. McCutchan and obtained from BEI Resources, NIAID, NIH. Parasites were propagated in donor ICR mice by the intraperitoneal inoculation of *P. berghei* ANKA from a frozen stock. Parasitemia was monitored by Giemsa-stained thin blood smears, and when it reached 20%–30%, blood was collected. For blood collection, mice were anesthetized with isoflurane until deep anesthesia was confirmed by the absence of reflexes (toe pinch and corneal reflex). Cardiac puncture was then performed, and euthanasia was completed by severing the cardiac apex, in accordance with OECD guidelines and institutional animal care regulations. All research procedures were conducted following the Ethical Guidelines for Animal Experimentation and Welfare established by the Research Institute for Health Sciences, Walailak University, Thailand (WU-ACUC-67062). The blood was processed and diluted with normal saline (0.9%) to achieve the standardized parasitic inoculum containing 1 × 10^7^ parasitized RBCs in 0.2 mL suspension. To evaluate the in vivo antimalarial activity against early infection, 30 mice received IP injection of 0.2 mL of 1 × 10^7^*P. berghei*–infected cells. Then, mice were randomly assigned to six experimental groups (five mice per group). Group 1 (Negative control group): Mice were orally administered a vehicle (7% Tween 80) to assess the baseline effects of the solvent. Groups 2 and 3 (Positive control groups): Mice orally administered 6 mg/kg ARS and 25 mg/kg CQ, respectively. Groups 4, 5, and 6 (Treatment groups): Mice were orally administered the selected crude extract at three different doses (200, 400, and 600 mg/kg). Treatment was begun at 3 h and then 24, 48, and 72 h postinfection. At the end of the experiment (Day 4), all mice were anesthetized with 2% isoflurane (Piramal Pharma, PA, USA) via inhalation using a rodent anesthetic system before being euthanized by heart puncture. Thin blood smears were made from blood samples to determine the percentage of parasitemia and the percentage of suppression. In addition, blood samples were used to determine the changes in hematological parameters, including RBC count, HCT, mean corpuscular volume (MCV), mean corpuscular hemoglobin (MCH), platelet count, and white blood cell (WBC) count. Hematologic measurements were performed using an AU480 automated chemistry analyzer (Beckman Coulter, USA). Parasitemia was monitored by Giemsa-stained thin blood smears, which were then viewed under a light microscope (Olympus CX31, Model CX31RBSFA, Tokyo, Japan) with oil immersion (100X magnification). The percentage parasitemia was calculated using the following formula [44]:

(5) $$\begin{array}{c} \%\text{ parasitemia}=\frac{\text{parasitized red blood cells}}{\text{total number of red blood cells}}\times 100. \end{array}$$

The percentage suppression of parasitemia in the treated groups relative to the negative control was calculated using the following equation:

(6) $$\begin{array}{c} \%\text{ parasitemia suppression}=\frac{\text{mean in negative control}-\text{mean in treatment group }}{\text{mean in negative control}}\times 100. \end{array}$$

### 2.14. Quantitative Assessment of Body Weight Changes in the 4-Day Suppressive Test

Body weights were measured using a high-precision digital analytical balance. The weights of individual mice were recorded on Day 0 (prior to infection) and again on Day 4 (post-treatment) at the end of the experimental period. The following formula was used to calculate the average percentage change in body weight for each experimental group [45]:

(7) $$\begin{array}{c} \%\text{ body weight change}=\frac{\text{body weight at Day }4-\text{body weight at Day }0}{\text{body weight at Day }4}\times 100. \end{array}$$

### 2.15. Assessment of Acute Oral Toxicity

An acute oral toxicity study was performed following OECD Guideline 425 to evaluate the safety of the selected crude extract. Ten male ICR mice were divided into two groups (*n* = 5): a control group receiving distilled water and a treatment group receiving a single dose of extract at 2000 mg/kg body weight. The crude extract was freshly prepared before dosing by dissolving the dried extract in distilled water to the required concentration. Control mice received the same volume of distilled water without the extract. Following body weight measurement, the treatment group received a single oral dose of 2000 mg/kg body weight via oral gavage. Mice were fasted for 2 h before dosing and observed for 14 days for signs of toxicity, including behavioral, neurological, gastrointestinal, and physiological changes. Additionally, daily food and water intake were recorded, and body weight was measured on Days 0 (pretreatment), 7, and 14 (post-treatment) using a sensitive digital analytical balance. At the end of the 14-day experimental period, the mice were humanely euthanized with 2% isoflurane (Piramal Pharma, PA, USA) via inhalation using a rodent anesthetic system. Adequate depth of anesthesia was confirmed using toe pinch and corneal reflex tests. After that, the blood was collected for the biochemical analysis of hepatic and renal function markers. The liver and kidney tissues were excised, rinsed in normal saline, and fixed in 10% paraformaldehyde for histopathological examination to detect potential cellular or structural damage. All animal handling, housing, and euthanasia procedures strictly complied with the guidelines and ethical standards established by the Walailak University Animal Care and Use Committee under the supervision of the Laboratory Animal Unit, Research Institute for Health Sciences, Walailak University, Thailand (WU-ACUC-67062).

### 2.16. Biochemical Analysis of Liver and Kidney Function

On Day 14 of the acute toxicity study, blood samples were collected for the biochemical evaluation of liver and kidney function. The collected blood was transferred into heparinized tubes to prevent coagulation and subsequently centrifuged at 3000 revolutions per minute (RPM) for 5 min to separate the plasma. The obtained plasma samples were subjected to biochemical analysis using an AU480 automated chemistry analyzer (Beckman Coulter, USA). The alanine aminotransferase (ALT), aspartate aminotransferase (AST), and alkaline phosphatase (ALP) were examined to detect liver function. Additionally, renal function indicators were evaluated by measuring the blood urea nitrogen (BUN) and serum creatinine levels. All assays were conducted in accordance with standard operating procedures (SOPs) to ensure the accuracy, reliability, and reproducibility of the results.

### 2.17. Evaluation of Cellular and Structural Alterations in Liver and Kidney Tissues

On day 14, following anesthesia and perfusion, the liver and kidney tissues were harvested from the experimental animals to investigate potential histopathological alterations. The excised organs were immediately fixed in 10% neutral buffered paraformaldehyde solution for a minimum of 24 h to preserve tissue morphology. The fixed tissues were subjected to standard histological processing. This included gradual dehydration through a series of increasing ethanol concentrations followed by clearing in xylene (three changes) to ensure the removal of lipids and facilitate tissue infiltration. After clearing, the tissues were embedded in molten paraffin wax to form paraffin blocks, which were then placed on a cooling plate for approximately 30 min for solidification. Once the blocks are hardened, tissue sections of 5 μm thickness were cut with a rotary microtome. The ribbons of the sections were flattened in a warm water bath, transferred onto clean glass slides, and allowed to dry thoroughly. For staining, tissue sections were deparaffinized with xylene (three changes, 10 min each) and rehydrated using an ethanol gradient (100%, 95%, 80%, and 70%). The slides were stained with hematoxylin and eosin (H&E) to visualize the cellular and tissue architecture. After staining, the sections were dehydrated using ascending ethanol concentrations, cleared with xylene, and mounted on cover slips using a suitable mounting medium. The prepared slides were examined under a light microscope by two independent, blinded histopathologists to assess and document any morphological abnormalities or pathological changes, such as cellular degeneration, necrosis, inflammation, or architectural disruptions in the liver and kidney tissues. This evaluation provides additional insights into the toxicological effects of the crude extract at the tissue level.

### 2.18. Statistical Analysis

All statistical analyses were carried out using the SPSS software (Version 29.0; IBM Corp., Armonk, NY, USA). Quantitative data are presented as the average ± standard deviation. Prior to further analysis, the normality of the data distribution for each parameter was evaluated using the Kolmogorov–Smirnov test. The half-maximal inhibitory concentration (IC_50_) and cytotoxic concentration (CC_50_) of each extract were determined by nonlinear regression analysis using GraphPad Prism Version 6 (GraphPad software, USA). One-way analysis of variance (ANOVA) was used to conduct comparative analyses between experimental groups, including parameters such as parasitemia percentage, parasitemia suppression percentage, food and water intake, body weight alterations, and biochemical indicators for the functioning of the kidneys and liver. Tukey's post hoc multiple comparison test was employed to assess intergroup variances. A statistical *p* value of less than 0.05 (*p* < 0.05) was considered statistically significant throughout the analysis. All the results were interpreted in the context of biological relevance and statistical significance.

## 3. Results

### 3.1. Percentage Yield of Crude Extracts From Phikud Navakot Formula and Its Components

The calculated yields are presented in Table 3. Among the formulations tested, the aqueous extract of *A. lancea* exhibited the highest extraction yield (52.67%), whereas the lowest extraction yield was recorded for the *N. jatamansi*, with a yield of only 14.31%. Notably, the aqueous extracts of *T. chebula* produced lower yields than the corresponding ethanolic extracts. In contrast, most of the remaining aqueous extracts yielded higher percentages than their ethanolic counterparts.

### 3.2. In Vitro Evaluation of the Antiplasmodial Activity

The in vitro antiplasmodial activities of the Phikud Navakot formula and its components are summarized in Table 4. According to established classification criteria for evaluating plant-derived antiplasmodial agents [46], extracts with an IC_50_ value less than 5 μg/mL are categorized as exhibiting potent activity, those with an IC_50_ between 5 and 15 μg/mL demonstrate good activity, IC_50_ between 15 and 50 μg/mL indicate moderate activity, and extracts with IC_50_ values greater than 100 μg/mL are considered inactive. Among the evaluated ethanolic crude extracts, *T. chebula* gall, *A. pallens* arial, *A. lancea* rhizome, and *N. jatamansi* rhizome exhibited good antiplasmodial activity with IC_50_ values of 4.39, 7.11, 9.58, and 10.96 μg/mL, respectively. The ethanolic extract of Phikud Navakot formulation showed moderate activity with an IC_50_ value of 15.64 μg/mL. The remaining ethanolic extracts demonstrated moderate antiplasmodial efficacy, with IC_50_ values ranging between 21.19 and 54.11 μg/mL. In contrast, most aqueous extracts exhibited limited or no antiplasmodial activity, with IC_50_ values generally exceeding 50 μg/mL. However, the aqueous extract of *T. chebula* gall exhibited potent activity, achieving an IC_50_ value of 3.24 μg/mL, while the aqueous extracts of Phikud Navakot formulation demonstrated moderate activity, with IC_50_ values of 18.37 μg/mL.

### 3.3. Evaluation of Cytotoxic Effects of Phikud Navakot Formula and Its Components

The assessment was conducted in accordance with the toxicity criteria established by the U.S. National Cancer Institute (NCI), which categorizes extracts with a CC_50_ value of less than 30 μg/mL, after 48–72 h of exposure, as cytotoxic. The finding revealed that most tested extracts exhibited CC_50_ values exceeding the cytotoxicity threshold, indicating relatively low toxicity toward Vero cells. Three ethanolic extracts—*L. sinense*, *S. costus,* and *A. pallens*—demonstrated mild cytotoxic effects on Vero cells, with CC_50_ values of 89.39, 63.17, and 61.02 μg/mL, respectively (Table 4).

### 3.4. SI

As summarized in Table 4, both aqueous and ethanolic extracts of *T. chebula* gall demonstrated exceptionally high SI values (> 20) in Vero cell lines, indicating strong antiplasmodial efficacy with low cytotoxic risk. Additionally, the ethanolic extract of *At. lancea* exhibited an SI greater than 10 in Vero cells, further suggesting a promising safety profile. Similarly, the ethanolic extracts of *N. jatamansi*, *A. pallens*, and Phikud Navakot showed elevated SI values in Vero cells.

### 3.5. Hemolysis of Phikud Navakot Formulation

**Figure 1:**
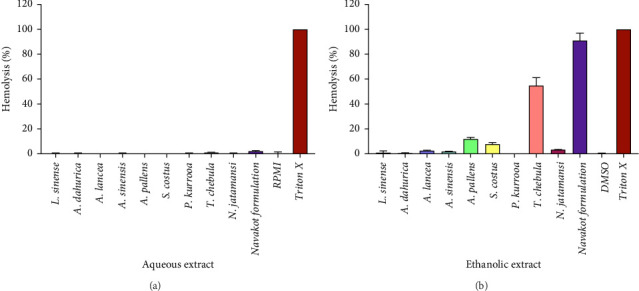
Percentage of hemolysis from in vitro hemolysis assay against human erythrocyte. (a) Hemolytic effects of aqueous extracts from Phikud Navakot formulation and its components at 50 μg/mL. (b) Hemolytic effects of ethanolic extracts from Phikud Navakot formulation and its components at 50 μg/mL. RPMI, Roswell Park Memorial Institute 1640 medium; and DMSO, dimethyl sulfoxide.

At a fixed concentration of 50 μg/mL, the aqueous extracts exhibited no significant hemolytic activity, indicating minimal or no cytotoxic effects on RBCs. In contrast, certain ethanolic extracts induced varying degrees of hemolysis. Notably, the Phikud Navakot ethanolic extract Phikud Navakot induced approximately 90% hemolysis after 72 h of incubation, suggesting substantial erythrocyte toxicity. Additionally, the extracts from *T. chebula* and *A. pallens* exhibited hemolysis rates of 54.90% and 12.02%, respectively (Figure 1).

### 3.6. Selection of Crude Extract as a Candidate for Mouse Model Evaluation

The selection of a promising crude extract for further in vivo testing in a mouse model was based on the assessment of the SI values of the 20 crude extracts, as presented in Table 4. Seven extracts did not yield SI values, including the aqueous extracts of *L. sinense*, *A dahurica*, *A. lancea, A. sinensis, A. pallens, S. costus,* and *P. kurrooa* for Vero cell lines, because of the inability to calculate the SI values. Among the tested extracts, the aqueous extract of *T. chebula* gall exhibited the highest (30.86) SI value, whereas the ethanolic extract demonstrated a consistently high SI value (22.77). The in vitro antiplasmodial activity of *T. chebula* gall extracts demonstrated comparable efficacy between the two solvent preparations. The aqueous extract exhibited an IC_50_ value of 3.24 ± 0.59 μg/mL, whereas the ethanolic extract presented a slightly higher IC_50_ value of 4.29 ± 2.28 μg/mL, suggesting that both extracts possess similar potency against *Plasmodium* spp. and are capable of effectively inhibiting parasite growth at low concentrations. In contrast, cytotoxicity assays conducted on Vero cells revealed that the ethanolic extract was more cytotoxic than the aqueous extract. Importantly, the aqueous extract displayed no detectable hemolytic activity, which is an essential consideration for any compound intended for systemic administration in antimalarial therapies. Considering its low cytotoxicity, absence of hemolytic activity, and favorable SI, the aqueous extract of *T. chebula* gall was identified as the most suitable candidate for further in vivo evaluation in mice models.

### 3.7. Chemical Characterization of the Aqueous Extract of *T. chebula* Gall

**Figure 2:**
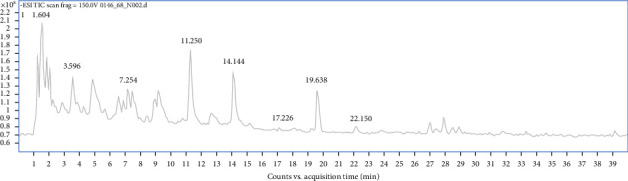
Full-scan chromatogram of *T. chebula* gall extract.

As summarized in Table 5, 53 distinct chemical constituents were detected in the aqueous extract of *T. chebula* galls, reflecting their diverse and rich phytochemical composition.  Figure 2 presents the chromatographic peak profiles of the aqueous extracts, offering a visual representation of the elution patterns and relative abundance of the detected metabolites.

### 3.8. Four-Day Suppressive Test of Antimalarial Activity

A summary of the parasitemia levels and percentage suppression across all treatment groups is presented in Table 6. The results demonstrated a dose-dependent suppression of parasitemia in mice treated with *T. chebula* gall extract. The *T. chebula* gall extract exhibited parasitemia suppression rates of 38.88%, 47.08%, and 60.61% at 200, 400, and 600 mg/kg, respectively. These suppressive effects were statistically significant when compared to the negative control group (*p* < 0.05). Treatment with *T. chebula* gall extract also reduced parasitemia levels compared to those in the negative control group, with the most pronounced effect observed at 600 mg/kg, indicating a potential therapeutic dose for further investigation. Although the crude extracts showed promising effects, they did not completely clear parasitemia. In contrast, the positive control drugs, ARS (6 mg/kg) and CQ (25 mg/kg), suppressed parasitemia by more than 80%; however, even the positive controls did not totally eradicate parasitemia.

### 3.9. Effect of *T. chebula* Gall Extract on Hematologic Changes in 4-Day Suppressive Test

**Figure 3:**
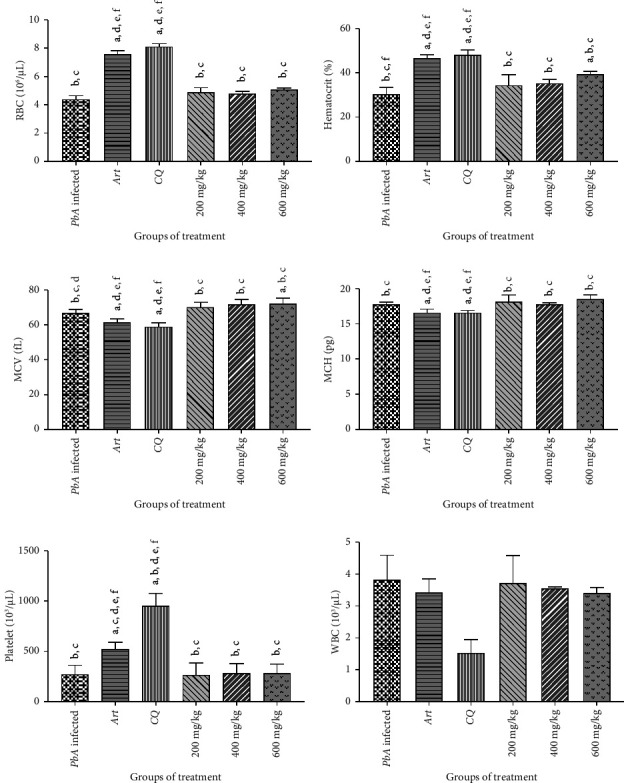
Hematological profiles of *Pb*A-infected and *Pb*A-treated mice. The measured parameters included red blood cell (RBC) count, hematocrit (HCT), mean corpuscular volume (MCV), mean corpuscular hemoglobin (MCH), platelet count, and white blood cell (WBC) count. The results are presented as mean ± standard deviation (SD) (*n* = 5). ^a^Compared to *Pb*A-infected, ^b^compared to standard artesunate, ^c^compared to standard chloroquine, ^d^compared to 200 mg/kg dose of the *T. chebula* gall extract, ^e^compared to 400 mg/kg dose of the *T. chebula* gall extract, ^f^compared to 600 mg/kg dose of the *T. chebula* gall extract. Differences were considered significant at *p* < 0.05.

On Day 4, the blood was collected from *Pb*A-infected control, standard antimalarial drug-treated (CQ and ARS), and groups treated with *T. chebula* gall extract at doses of 200, 400, and 600 mg/kg body weight in order to study hematologic changes. The hematologic parameters of *P. berghei* ANKA–infected and *P. berghei* ANKA–treated mice are shown in Figure 3. Specifically, significant differences (*p* < 0.05) were noted in the HCT and MCV values between the infected and treatment groups, indicating the hematologic impact of parasitic infection. Although the changes in RBC counts, MCH, platelet, and WBC counts were observed across the groups, these variations did not reach statistical significance in certain comparisons. Treatment with the standard antimalarial drug led to a significant increase in the RBC count, HCT, and platelet count (*p* < 0.05) compared to those in the infected control group. In contrast, this group exhibited a marked reduction in the MCV and MCH levels (*p* < 0.05). Among the extract-treated groups, mice receiving the *T. chebula* gall extract at doses of 200, 400, and 600 mg/kg showed an increase in the RBC count and HCT compared to the *P. berghei* ANKA–infected group. Furthermore, the analysis of MCV and MCH values demonstrated that all doses of *T. chebula* gall extract resulted in significantly higher MCV and MCH levels (*p* < 0.05) compared to the *P. berghei* ANKA–infected and antimalarial standard drug groups. Platelet counts were significantly reduced (*p* < 0.05) in *P*. *berghei* ANKA–infected mice, indicating that thrombocytopenia was associated with malarial infection. Notably, only the standard drug-treated group exhibited a significant restoration of the platelet count (*p* < 0.05) compared to the *P. berghei* ANKA–infected group. Additionally, ARS and CQ treatment resulted in a significant reduction in WBC counts (*p* < 0.05) compared to that in the infected group. *T. chebula* gall extract treatment did not significantly prevent WBC depletion compared to the standard antimalarial drug. However, WBC counts were reduced (*p* < 0.05) in mice treated with *T. chebula* gall extract at 200, 400, and 600 mg/kg doses compared to those in the infected control.

### 3.10. Effect of *T. chebula* Gall Extract on Bodyweight Changes in 4-Day Suppressive Test

Mice infected with *P. berghei* ANKA demonstrated a progressive and significant decline in body weight, with an average reduction of approximately 0.68% relative to their baseline weight. In contrast, by Day 4 postinfection, mice that received antimalarial treatment with ARS and CQ exhibited marked improvements in body weight, with increases of 9.37% and 6.96%, respectively, compared to their initial baseline values. Likewise, mice administered the aqueous crude extract at doses of 400 and 600 mg/kg showed moderate increases in the body weight of 1.63% and 2.93%, respectively. Statistical analysis revealed that the treatment groups experienced greater weight gain than the untreated group (*p* < 0.05) (Table 7).

### 3.11. Assessment of Physical Activity, Behavioral Observations, Food and Water Intake, and Body Weight in Acute Toxicity Test of *T. chebula* Gall Extracts

Throughout the observation period, no adverse clinical signs or behavioral abnormalities were observed in any treatment group. Specifically, there were no manifestations of toxicity, such as tremors, rigidity, ataxia, altered grooming behavior, piloerection, abnormal posture, changes in sleep patterns, diarrhea, vomiting, altered feeding or drinking behavior, or changes in locomotor activity. Moreover, no mortality was observed within the initial 24 h or during the 14-day period in the extract-treated groups. Statistical analysis revealed no significant differences in food and water intake between the extract-treated groups (2000 mg/kg *T. chebula* gall extract) and the control group (*p* > 0.05), as shown in Table 8. Additionally, body weight measurements recorded at the beginning (average of Week 1) and end (average of Week 2) of the study demonstrated no statistically significant differences between the treatment and control groups (*p* > 0.05), as presented in Table 9.

### 3.12. Liver and Kidney Function Biochemical Parameters

Biochemical analyses of liver and kidney function markers were performed at the end of the 14-day acute toxicity study, as presented in Table 10. The levels of liver function enzymes—including AST, ALT, and ALP—in mice administered a single oral dose of 2000 mg/kg body weight *T. chebula* gall extract showed no significant differences compared to those of the untreated control group (*p* > 0.05). Additionally, kidney function parameters, including BUN and creatinine levels, remained within the normal physiological range [47] across the treatment groups. No statistically significant differences (*p* > 0.05) were observed between mice treated with the crude extract and those in the untreated control group. These findings suggest that aqueous crude extracts of *T. chebula* galls do not exert hepatotoxic or nephrotoxic effects at a dose of 2000 mg/kg body weight under the conditions of this study.

### 3.13. Histopathological Examination of Liver and Kidney Tissues

**Figure 4:**
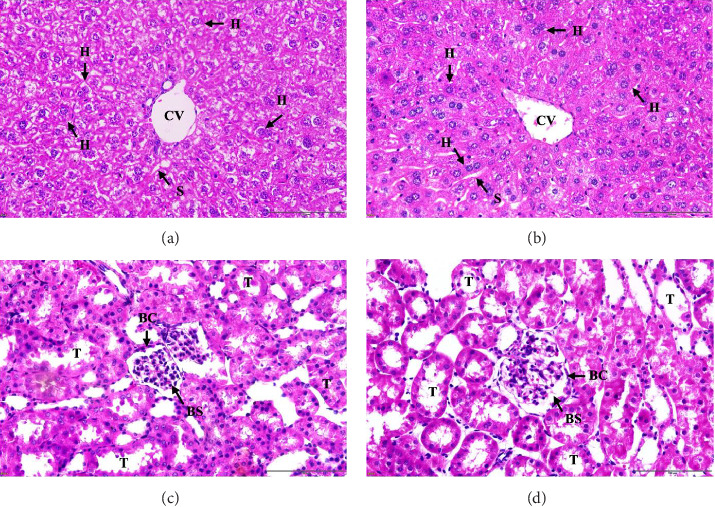
Histopathological examination of liver and kidney tissues following an acute oral toxicity test. Panels (a) and (c) depict the tissue samples from the control group, while panels (b) and (d) show the samples from the experimental group administered *T. chebula* gall extract at a dosage of 2000 mg/kg body weight. All tissue sections were stained with hematoxylin and eosin (H&E) and imaged at 20x magnification, with a scale bar of 200 μm. Key histological structures identified include the central vein (CV), hepatocytes (H), sinusoidal capillaries (S), glomerulus (G), Bowman's capsule (BC), Bowman's space (BS), and renal tubules (T).

Representative micrographs of the histological sections are shown in Figure 4. Liver sections from mice treated with a single dose of 2000 mg/kg body weight *T. chebula* galls displayed a well-preserved hepatic architecture. The hepatocytes appeared morphologically normal, characterized by a polygonal cell shape, eosinophilic cytoplasm, and centrally located round nuclei. The hepatic sinusoids, portal triads, and central veins were structurally intact and showed no evidence of vascular congestion, sinusoidal dilatation, cellular degeneration, or inflammatory cell infiltration. Similarly, renal tissues exhibited normal histological features. The glomeruli were well-defined, with no indications of glomerular hypertrophy, atrophy, or mesangial expansion. Bowman's capsules remained intact, and renal tubular epithelial cells retained their normal morphology without evidence of cytoplasmic vacuolization, tubular necrosis, or interstitial fibrosis. These findings are consistent with the observations in the control group.

## 4. Discussion

The results of this study indicate that among the 20 crude extracts tested, the *T. chebula* gall extract exhibited the most potent in vitro antimalarial activity against the *P. falciparum* K1 strain, with an IC_50_ value of 3.24 ± 0.59 μg/mL. Previous research has shown several pharmacological activities, including antimicrobial, anti-inflammatory, antioxidant, and cytotoxic properties [48]. The presence of such phytochemicals may play a crucial role in disrupting malarial parasite survival mechanisms such as oxidative stress regulation and metabolic pathways essential for parasite replication [49]. Moreover, the antimalarial potential observed in *T. chebula* (seeds and fruit) could be attributed to its well-documented phytochemical constituents, including chebulagic acid, chebulinic acid, and gallic acid [50]. A previous study found that *T. chebula* fruit extract had IC_50_ values of 5.05 ± 1.86 and 4.53 ± 0.82 μg/mL for the ethanolic and aqueous extracts, respectively [33]. The elevated antioxidant capacity of certain phytochemicals can significantly disrupt the redox balance within the *Plasmodium* parasite, leading to enhanced oxidative stress and damage to the cellular structure. Studies have demonstrated that compounds capable of modulating parasite redox homeostasis can interfere with essential cellular functions, ultimately impairing parasite survival [51].

The assessment of the hemolytic activity of the Phikud Navakot formulation and its components offers crucial insights into their potential hematotoxic effects on human erythrocytes. Hemolysis, characterized by the rupture of RBCs and the subsequent release of hemoglobin into the surrounding plasma, serves as a fundamental indicator of erythrocyte cytotoxicity. This evaluation is particularly significant in the context of herbal formulations because certain plant-derived compounds can compromise the integrity of erythrocyte membranes, leading to hemolysis. Therefore, assessing hemolytic activity is essential for determining the safety and biocompatibility of herbal products intended for therapeutic use [52]. At a standardized concentration of 50 μg/mL, the aqueous extracts of the Phikud Navakot formulation and its individual components demonstrated no significant hemolytic activity. This finding suggests that these extracts are unlikely to induce substantial erythrocyte damage under the experimental conditions. The absence of notable hemolysis indicates that the aqueous extracts exhibit minimal or no cytotoxic effects on human RBCs, reinforcing their potential safety in therapeutic applications. Furthermore, this observation is consistent with previous studies, which reported that Phikud Navakot extract, even at concentrations as high as 1 mg/mL, did not cause significant hemolysis in sheep erythrocytes [12]. These findings collectively support the hypothesis that aqueous extracts of the Phikud Navakot formulation maintain low hemolytic potential, further validating their suitability for medicinal use. Contrastingly, the ethanolic extracts demonstrated varying degrees of hemolysis, with the ethanolic extract of the Phikud Navakot formulation being particularly noteworthy. It induced approximately 90% hemolysis after 72 h of incubation, indicating substantial erythrocyte toxicity. This significant hemolytic activity raises concerns regarding the potential hematotoxicity of the formulation, suggesting that it could pose a risk to RBC integrity if used in vivo or under certain conditions. The ethanolic extract of *T. chebula* demonstrated a hemolysis rate of 54.90%, indicating significant potential for erythrocyte membrane disruption. This observation is consistent with a previous study, which has reported that Phikud Navakot formulation and *T. chebula* contain a high abundance of bioactive compounds, particularly flavonoid and alkaloid, that may compromise erythrocyte membrane integrity and consequently induce hemolysis [30, 53]. The result is consistent with the findings of Mapfununde et al., which reported that alkaloids exhibited the highest hemolytic activity. The alkaloid-enriched fraction show a value of 0.286 mg/mL [54]. In addition, the ethanol extract of *T. chebula* was very toxic to brine shrimps. The ethanol extract had the highest total phenolic and flavonoid contents of 136 ± 1.5 mg of gallic acid equivalent/g d.w and 113 ± 1.6 mg of quercetin equivalent/g d.w, respectively. The higher toxicity effect was positively correlated to the high content of total polyphenols/flavonoids in the extract [55]. These results suggested that the ethanolic extract of the formulation, particularly the combination of *T. chebula* and other components, may require further refinement to reduce its hemolytic potential. The high degree of hemolysis observed in the Phikud Navakot formulation warrants caution in its therapeutic application, particularly in formulations intended for oral or intravenous administration, where the potential for RBC destruction could lead to adverse effects such as anemia or hemolytic reactions [56]. These findings emphasize the need for further investigation into the safety and formulation optimization of Phikud Navakot, with a particular focus on reducing its hemolytic activity to ensure its safe use in clinical settings.

All of the crude extracts tested for cytotoxicity were found to be nontoxic, with CC_50_ values more than 30 μg/mL, indicating a good safety profile [57]. Notably, extracts with higher SI values, particularly those over 10, were considered more promising because of their strong specificity against *P. falciparum*, corresponding with the established standards for possible antimalarial candidates [58]. Consequently, we evaluated the cytotoxic effects of crude extracts on Vero cells. Among the 20 crude extracts assessed, *T. chebula* gall satisfied the approval criteria by having SI values greater than 10, showing substantial therapeutic potential. Furthermore, these extracts exhibited notable antimalarial activity based on IC_50_ value classifications, while maintaining a nontoxic profile against Vero cells. Based on these findings, *T. chebula* gall extracts were identified as the most promising candidates and chosen for further in vivo studies to assess their antimalarial activity and acute oral toxicity in mouse models.

In the present investigation, a 4-day suppressive test was used to assess the antimalarial activity of *T. chebula* gall extracts against *P. berghei* ANKA infection in an ICR mouse model. The results showed that all groups of mice treated with the aqueous extracts had dose-dependent suppression of *P. berghei* ANKA, with significantly lower parasitemia levels than the negative control group. The group that received the greatest dose of *T. chebula* gall extract (600 mg/kg body weight) had the highest suppression rate among all treatment groups, achieving a parasitemia suppression of 60.00% relative to the negative control group. Despite the promising efficacy of the aqueous extracts, mice treated with the extracts exhibited significantly lower suppression rates than those treated with standard antimalarial drugs, specifically ARS (80%) and CQ (98%). This lowered suppression could be attributable to a lower concentration or bioavailability of the therapeutic chemicals in the extracts, which could affect their pharmacokinetics and overall efficacy. However, compounds with a parasitemia suppression rate of ≥ 30% compared to the negative control group have antimalarial potential, according to recognized criteria. The literature assesses a compound's antimalarial activity as very good, moderate, or good based on doses that limit growth by 50% or more. This assignment is graded as follows: extremely good (≤ 100 mg/kg/day), good (250-100 mg/kg/day), and moderate (500-250 mg/kg/day) [59]. Based on these criteria, all dosages of aqueous extracts met the threshold, confirming their schizonticidal activity against *P. berghei* in ANKA-infected mice. This result is in line with another study that found that *T. chebula* fruit extract, administered at a dose of 250 mg/kg/day, had high suppression activity. The treated mice showed a mean parasitemia level of 12.07 with 68.89% suppression [60]. Moreover, the parasitemia suppression in this study is superior to the study done on the *Sesamum indicum* [61], *Ocimum lamiifolium* [62], *Olea europaea* [63], and *Vernonia amygdalina* [64]. The observed antimalarial effects of *T. chebula* crude extracts may be attributed to their rich phytochemical composition, which includes polyphenols, flavonoids, alkaloids, terpenoids, and saponins [65]. These findings are in accordance with those of previous studies suggesting that numerous secondary metabolites—including alkaloids, terpenes, flavonoids, xanthones, anthraquinones, phenols, and sesquiterpenes—have antimalarial effects [66]. Specific phenolic compounds have been identified to have substantial action against *P. falciparum*. Research on *Alectryon serratus* leaves found gallic acid, methyl gallate, and kaempferol-3-O-rhamnoside with IC_50_ of 0.0722, 0.0128, and 3.4595 μM, respectively [67]. The antimalarial efficacy of phenolic compounds is linked to many processes such as the production of ROS that harm the parasite, blocking essential enzymes required for parasite survival, and interfering with the capacity of the parasite to detoxify heme [68]. A previous study evaluated eight naturally occurring dietary flavonoids and their analogs for their antiplasmodial activity against clinical isolates of *P. falciparum* in southeastern Bangladesh. The results showed that most of these flavonoids had IC_50_ below 14 μM, indicating notable antimalarial effects [69]. A previous study reported that commercial drugs containing flavonoids were active in mice with malaria (63% suppression) and exhibited activity against CQ-resistant *P. falciparum* in vitro (IC_50_ 5 ± 3.9 μg/mL) [70]. Flavonoids may interfere with enzymes crucial for parasite metabolism and survival. Molecular docking studies have suggested that flavonoids can bind to antimalarial targets and potentially inhibit their function [71]. Another key mechanism is the inhibition of heme detoxification. During hemoglobin digestion by the parasite, toxic free heme is released and normally detoxified into an inert crystalline form known as hemozoin. Certain flavonoids can inhibit this process, leading to the accumulation of toxic heme within the parasite, ultimately resulting in cell death [72]. Specific triterpenoids have demonstrated the ability to inhibit the growth of *P. falciparum*. For instance, studies on *Diospyros rubra* have identified compounds such as lupeol, lupenone, and betulin that exhibit notable antimalarial activity [73]. As a result, the chemical mentioned above, as well as the other compounds found in extract, may have antimalarial properties either individually or synergistically. However, further experimental validation, compound isolation, and mechanistic studies are required to advance its development as an alternative or complementary therapy against malaria.

In this study, the hematologic parameters of *P. berghei* ANKA–infected and treated mice were investigated, revealing significant alterations compared to the treatment groups. A key observation in this study was the significant differences in HCT, MCV, and MCH between the infected and treatment groups, highlighting the hematologic disruption caused by infection. Specifically, the reduction in MCV and MCH in the infected mice suggested microcytic hypochromic anemia, a common feature of malaria, due to the destruction of RBCs and impaired erythropoiesis. These results are in line with classic malaria-induced anemia caused by the sequestration of infected erythrocytes and the body's compensatory mechanisms to produce new blood cells to counteract blood loss [74]. Interestingly, treatment with the standard antimalarial drugs ARS or CQ significantly increased RBC counts (*p* < 0.05) compared to those in the infected control group. This increase in the RBC count, along with the reduced MCV and MCH levels, implies a partial recovery of erythropoiesis, suggesting that antimalarial treatment facilitates the restoration of normal erythrocyte morphology and function. The significant reduction in MCV and MCH following treatment with standard antimalarial drugs could indicate the rapid restoration of RBC integrity after parasitic clearance, as the body attempts to normalize the RBC population [75]. Among the extract-treated groups, *T. chebula* gall extracts administered at doses of 200, 400, and 600 mg/kg showed a positive effect on RBC counts, with significant increases compared to the infected group. Furthermore, the analysis of MCV and MCH values demonstrated that all doses of *T. chebula* gall extract resulted in higher MCV and MCH values (*p* < 0.05) than those in both the infected control and antimalarial drug-treated groups. The observed improvement in hematological parameters after treatment with *T. chebula* gall extract appears to result from effective parasite clearance, suggesting that the restoration of RBC levels is a downstream consequence of its antiplasmodial activity. A significant reduction in the platelet count was observed in *P. berghei* ANKA–infected mice, indicating thrombocytopenia, a common complication of malaria infection [76]. Interestingly, only the standard antimalarial drug-treated group exhibited a significant restoration of platelet count compared with the infected control group, reflecting its efficacy in mitigating malaria-induced hematological complications [77]. However, *T. chebula* gall extract treatment did not show a significant restoration of platelet counts. This could be due to the different mechanisms of action between the extract and the standard drugs. The extract may exert a weaker antiparasitic effect compared to the standard treatment, resulting in less effective parasite clearance and consequently slower or incomplete restoration of platelet counts. Additionally, significant reductions in WBC counts were observed in the ARS- and CQ-treated groups compared to the infected group. This reduction could be attributed to the immunosuppressive effects of these drugs. In contrast, *T. chebula* gall extract treatment did not significantly prevent WBC depletion compared to standard antimalarial drugs. However, a dose-dependent reduction in WBC counts was observed in the extract-treated groups compared to the infected control, suggesting that the *T. chebula* gall extract may modulate immune cell populations, possibly by modulating the inflammatory response or influencing the activity of immune cells during malaria infection [78]. These results suggest that *T. chebula* gall extract improves hematological parameters by effectively suppressing parasitemia, indicating that parasite clearance contributes to the recovery of RBC function in malaria-induced anemia.

Malaria-induced weight loss is a well-documented consequence of parasitic infections, primarily resulting from systemic inflammation, metabolic dysregulation, and increased energy expenditure owing to immune activation [79]. In this study, mice infected with *P. berghei* ANKA exhibited a steady and significant decline in body weight, with an average reduction of approximately 0.68% relative to baseline weight. This progressive weight loss is indicative of the severe physiological stress and cachexia associated with malaria, wherein parasitemia leads to metabolic exhaustion, reduced food intake, and muscle catabolism. In contrast, mice that received the antimalarial treatment with ARS and CQ showed a notable improvement in body weight. By Day 4 postinfection, ARS-treated mice exhibited a weight gain of 9.37%, whereas CQ-treated mice exhibited a 6.96% increase. These results are consistent with those of previous studies that demonstrated the efficacy of standard antimalarial drugs in reducing parasitemia and mitigating malaria-associated weight loss and systemic inflammation [80]. The observed weight restoration can be attributed to the ability of the drug to rapidly clear parasites, thereby preventing further metabolic deterioration and promoting recovery [77]. Similarly, the administration of the aqueous crude extract at doses of 400 and 600 mg/kg resulted in moderate increases in body weight by 1.63% and 2.93%, respectively. This result consistent with previous study found that the crude extracts such as *Lagenaria siceraria* [59], *Commelina latifolia* [81], and *Croton macrostachyus* [82] prevent weight loss in *P. berghei*–infected mice. Although the weight gain observed in the extract-treated groups was less pronounced than that observed in the ARS- and CQ-treated groups, it suggests a partial attenuation of infection-induced cachexia. This finding implies that the plant extract may possess antimalarial or immunomodulatory properties that contribute to maintaining metabolic balance and reducing the adverse effects of infection [83]. Previous studies have demonstrated that *T. chebula* extracts enhance antioxidant and anti-inflammatory capabilities, improve growth performance, and promote intestinal health in animal models. Additionally, *T. chebula* has been shown to regulate pro-inflammatory and anti-inflammatory cytokine levels and adjust antioxidant indicators in mice [84]. Furthermore, the extract exhibited analgesic and anti-inflammatory effects in experimental animal models [85]. Overall, these findings highlight the importance of maintaining nutritional and metabolic homeostasis in malaria-infected individuals and underscore the need for effective therapeutic interventions that not only target parasitic clearance but also mitigate secondary complications such as weight loss and cachexia. The further research is required to elucidate the specific biochemical pathways involved in weight restoration and determine the precise mode of action of the extract.

This study employed LC-MS to analyze the chemical constituents of *T. chebula* gall extracts. We found that *T. chebula* gall extracts contain chebulic acid (phenolic), Punicacortein D (phenolic), amlaic acid, isoterchebin (polyphenols), chebulinic acid (polyphenols), rhamnocitrin 3-(6″-acetylglucoside) (flavonoids), sergeolide (triterpenoid), luteolin (flavonoids), and Ganolucidic Acid A (triterpenoids). Chebulic acid, a hydrolyzable tannin predominantly found in *T. chebula*, has attracted scientific attention for its potential antimalarial activity. This compound exhibits multiple pharmacological effects, including antioxidant, anti-inflammatory, and antimicrobial properties, which may contribute to its antiplasmodial efficacy [86]. Chebulic acid exhibits iron-chelating properties that may contribute to its therapeutic potential [87]. Iron is crucial for *Plasmodium* survival and serves as a cofactor in vital processes, such as DNA synthesis via ribonucleotide reductase and mitochondrial electron transport [88]. Its iron-chelating properties may inhibit parasite development by sequestering iron within infected erythrocytes, leading to impaired DNA synthesis and reduced parasite proliferation [89]. Chebulic acid also exhibits free radical-scavenging abilities [90], which can mitigate the oxidative stress and tissue damage associated with malarial infection, potentially enhancing host recovery and reducing inflammation. Furthermore, its synergistic interaction with other polyphenols and antimalarial agents may enhance therapeutic outcomes [91]. These findings support the pharmacological relevance of chebulic acid as a promising natural compound for adjunct or alternative malaria therapies. Sergeolide, a quassinoid compound isolated from *Picrolemma pseudocoffea*, demonstrates potent antimalarial activity both in vitro and in vivo studies. In laboratory experiments, sergeolide exhibited strong inhibitory effects against *P. falciparum*, including CQ-resistant strains. Complete inhibition of parasite growth was achieved at concentrations as low as 0.006 μg/mL, with significant effects observed even at 0.002 μg/mL. Sergeolide was effective against both CQ-sensitive and CQ-resistant strains, highlighting its broad-spectrum potential. In animal models, specifically mice infected with *P. berghei*, sergeolide significantly reduced parasitemia [92]. Luteolin, a flavonoid commonly found in various plants, such as celery, broccoli, green pepper, and parsley, has demonstrated notable antimalarial properties in several studies [93]. Luteolin effectively inhibits the growth of *P. falciparum*, the parasite responsible for malaria. Luteolin exhibited IC_50_ values of approximately 11 and 12 μM against CQ-sensitive (3D7) and CQ-resistant (7G8) strains of *P. falciparum*, respectively. This compound arrests parasite development at the young trophozoite stage, preventing progression to later stages of the parasite life cycle [94]. Further investigations identified luteolin in various plant extracts as having antiplasmodial activity. For instance, a study on *Citrus maxima* root extract isolated luteolin as one of the active compounds, showing IC_50_ values of 2.315 ± 0.489 and 2.691 ± 0.454 μg/mL against CQ-sensitive (*Pf*3D7) and CQ-resistant (*Pf*RKL-9) strains, respectively [95]. The antimalarial mechanism of luteolin has not been fully elucidated but is believed to involve multiple pathways. Molecular docking studies suggested that luteolin may inhibit key enzymes in *P. falciparum*, such as lactate dehydrogenase (*Pf*LDH) and enoyl-ACP reductase (*Pf*ENR), which are crucial for parasite metabolism and survival [95]. Luteolin has been associated with the inhibition of fatty acid biosynthesis in parasites, impairing the development of vital organelle components necessary for parasite growth [96]. Gallic acid acts by generating oxidative stress within the parasite, disrupting its metabolic pathways and thereby suppressing its growth [97]. These constituents are known to exert pharmacological effects such as oxidative stress induction, inhibition of heme polymerization, interference with parasitic enzymatic pathways, and immunomodulation, all of which are relevant to antiplasmodial action. These findings imply that the antimalarial efficacy of the extract is likely attributable to the combined or synergistic interactions of these phytochemicals, rather than to a single active compound. This multifaceted mode of action enhanced the therapeutic value of the extract and offered a promising natural alternative for malaria control.

The acute toxicity test is an important component of preclinical safety assessments that evaluates the potential short-term side effects of a single high-dose administration of a test chemical. This test is usually performed in the early stages of drug development to create preliminary safety profiles, estimate dose thresholds, and identify probable toxicological symptoms [98]. The data obtained from these studies provided essential insights into the potential toxicity of the substance, supporting further pharmacological evaluations and guiding subsequent dosing strategies in long-term studies. One of the key physiological systems affected by toxic substances is the hematopoietic system. Because hematopoietic tissues are highly sensitive to toxic insults, hematologic parameters serve as valuable biomarkers for assessing systemic toxicity and predicting potential adverse effects in both human and animal models [99]. Beyond hematologic indices, the liver and kidneys are the primary organs responsible for the metabolism, detoxification, and removal of xenobiotics, including pharmaceutical substances [100]. Hepatic toxicity is commonly assessed by evaluating the levels of liver marker enzymes such as AST, ALT, and ALP. These enzymes are released into systemic circulation following hepatocellular damage, making them critical indicators of liver function and integrity [101]. Similarly, renal function is a key parameter in toxicity evaluation, because the kidneys play a fundamental role in filtering and excreting endogenous and exogenous substances [102]. Overall, acute toxicity studies provided critical preliminary data regarding the potential systemic effects of new compounds. The integration of hematologic, hepatic, and renal biomarkers in these assessments enhances the reliability of toxicity profiling, facilitating the identification of safe dosage levels and providing risk-benefit analyses in drug development.

In the present study, mice received a single oral dose of *T. chebula* gall extract at 2000 mg/kg body weight and showed no evidence of acute toxicity. During the 14-day post-treatment observation period, no mortality or treatment-related adverse effects were seen, indicating that the extract is well tolerated at this dosage and may be regarded as nontoxic under the parameters of this investigation. Furthermore, biochemical analyses of plasma markers associated with liver and kidney function revealed no significant differences between the extract-treated and control groups, specifically in the levels of AST, ALT, ALP, and creatinine. There was a statistically significant increase in BUN levels in the extract-treated groups compared to those in the normal group. Elevated BUN levels are commonly regarded as a biochemical indicator of potential renal dysfunction, particularly in preclinical toxicity assessments [103]. However, in this study, the observed BUN values remained within the established physiological range in mice, suggesting that the *T. chebula* gall extract did not induce nephrotoxic effects under the administered conditions. This is consistent with previous studies demonstrating the overall safety of *T. chebula* extracts in various animal models, where no adverse effects on renal or hepatic function were observed, even at relatively high doses. From previous study, a single oral dose of *T. chebula* extract at 5000 mg/kg showed no acute toxicity in rats. In the chronic study, daily oral administration at 300, 600, and 1200 mg/kg for 270 days caused no abnormalities compared to controls. No changes were observed in behavior, mortality, body weight, organ weight, hematological or biochemical parameters, or histopathology. These findings indicate that *T. chebula* is nontoxic under the tested conditions [104]. Thus, the data support the conclusion that *T. chebula* gall extract does not exert nephrotoxicity at the tested dosages.

Histopathological examination further corroborated these biochemical findings, demonstrating that the liver and kidney tissues from the extract-treated mice maintained normal morphological integrity. The absence of structural alterations in hepatocytes and renal tubules suggests that the extracts did not induce histopathological damage [105]. Changes in body weight are commonly used as indicators of the potential toxic effects of test substances, as adverse reactions to compounds often manifest as reduced appetite and metabolic disturbance [106]. The results of this study demonstrated that mice treated with *T. chebula* gall extract exhibited no significant differences in body weight compared to the control groups. Moreover, all groups, including the extract-treated and control mice, displayed consistent weight gain throughout the experimental period. This suggests that the extract does not negatively affect food or water intake, supporting its potential safety in terms of metabolic effects. Additionally, food and water consumption behaviors in the extract-treated mice remained comparable to those in the control groups, reinforcing the absence of adverse physiological responses to the administered dose. Based on these findings, the estimated median lethal dose (LD_50_) of *T. chebula* gall extract was determined to be greater than 2000 mg/kg body weight when administered orally as a single dose. The Globally Harmonized System of Classification and Labelling of Chemicals (GHS) classifies compounds with LD_50_ above this threshold as Class 5, signifying moderate toxicity and a low risk of acute adverse effects [107]. Although the results of this study support the safety of *T. chebula* gall extracts at high doses, further toxicological evaluations are necessary to establish comprehensive safety profiles. Subacute and chronic toxicity studies should be conducted to assess the long-term effects of repeated administration, pharmacokinetic properties, and potential cumulative toxicity before progressing to human clinical trials.

## 5. Conclusions

This study presents the first comprehensive evidence that *T. chebula* gall extract has both in vitro antiplasmodial activity against CQ-resistant *P. falciparum* K1 and in vivo efficacy against *P. berghei* ANKA. The aqueous extract of *T. chebula* gall showed activity without cytotoxic or hemolytic effects, highlighting its biosafety. In mice model, the aqueous extract of *T. chebula* gall at a dose of 600 mg/kg achieved the highest parasitemia suppression in mice, suggesting dose-dependent efficacy. Additionally, *T. chebula* gall extract did not induce signs of acute toxicity in the mouse models when administered orally at a single dosage of 2000 mg/kg body weight.

## Figures and Tables

**Table 1:** Botanical constituents and pharmacological profile of the Phikud Navakot formula and its component.

Plant components	Common name	Part used	Family	Pharmacological properties	Voucher number
*L. sinense*	Kot Hua Bua	Root	Apiaceae	Anti-Alzheimer, anti-Parkinson, anti-inflammation, anticancer, analgesia, contraction inhibition, angiogenesis inhibition, and neuroprotection effect [19]	SMD017003002
*A. dahurica*	Kot soa	Root	Apiaceae	Antioxidant, anti-inflammatory, antitumor, wound healing, nervous system, antibacterial, and antivirus activities [20]	SMD276002003
*A. lancea*	Kot Khamao	Rhizome	Compositae	Anti-inflammatory, antioxidant, antivirus, antitumor, liver protection [21], anticancer, antiobesity [22], antiplasmodial [10]	SMD072010001
*A. sinensis*	Kot Chiang	Root	Apiaceae	Antitumor, anemia-enhancing, anti-inflammatory, antioxidant, immunomodulatory, hepatoprotective, antifibrotic, hypoglycemic, radioprotective, and antiviral effects [23], inhibition of platelet aggregation, antibacterial, and antiviral [24].	SMD017003003
*A. pallens*	Kot Chula-lumpa	Aerial part	Compositae	Antioxidant, anti-inflammatory, and antimicrobial effects [25], analgesic, antipyretic [26], anticancer, antidiabetic, antibacterial, antiplasmodial, and antifungal [27].	SMD024008001
*S. costus*	Kot Kradook	Rhizome	Compositae	Anti-inflammatory, antioxidant, anticancer, hepatoprotective, and immunomodulatory effects [28]	SMD029029001
*P. kurrooa*	Kot kan Prao	Rhizome	Plantaginaceae	Hepatoprotective, antioxidant, anti-inflammatory, anticancer, immunomodulator, anti-ulcerative colitis, and antimicrobial [29]	SMD021216001
*T. chebula*	Kot Phung Pla	Gall	Combretaceae	Antioxidant property, antiaging activity, anti-inflammatory, antidiabetic activity, cardioprotective activity, antifungal [30], typhoid fever [31].	SMD027074001
*N. jatamansi*	Kot Jatamansi	Root and rhizome	Caprifoliaceae	Antifungal, antimicrobial, antioxidant, hepatoprotective. and cardio protective properties [32]	SMD010057001

**Table 2:** Dosage in antimalarial activity assay.

Group of mice	Stock solution	Dose (mg/kg)	Orally administered volume (μL)
Negative group	7% Tween 80	—	200
Positive Group 1	Artesunate	6	200
Positive Group 2	Chloroquine	25	200
Experimental group	Selected crude extract from Phikud Navakot formulation	200	200
		400	200
		600	200

**Table 3:** Percentage extraction yields of aqueous and ethanolic extracts of Phikud Navakot formula and its components.

Crude extract	Yield (%) (w/w)	
	Aqueous extract (%)	Ethanolic extract (%)
*L. sinense*	33.57	9.49
*A. dahurica*	26.86	4.54
*A. lancea*	52.67	22.11
*A. sinensis*	38.08	5.72
*A. pallens*	27.57	6.63
*S. costus*	50.10	12.88
*P. kurrooa*	36.70	27.85
*T. chebula*	45.01	58.76
*N. jatamansi*	14.31	5.66
Phikud Navakot formula	39.20	17.18

**Table 4:** Antimalarial activity, cytotoxicity, and SI values of the Phikud Navakot formulation and its components.

Crude extract	Aqueous extract			Ethanolic extract		
	IC_50_ μg/mL	CC_50_ μg/mL	SI value	IC_50_ μg/mL	CC_50_ μg/mL	SI value
*L. sinense*	> 100	> 100	> 1.00	21.19 ± 0.63	89.39 ± 25.53	4.21
*A. dahurica*	> 100	> 100	> 1.00	39.71 ± 6.76	> 100	> 2.51
*A. lancea*	> 100	> 100	> 1.00	9.58 ± 2.73	> 100	> 10.43
*A. sinensis*	> 100	> 100	> 1.00	44.49 ± 13.07	> 100	> 2.24
*A. pallens*	> 100	> 100	> 1.00	7.11 ± 0.74	62.95 ± 15.57	8.85
*S. costus*	> 100	> 100	> 1.00	28.56 ± 1.47	63.17 ± 4.97	2.21
*P. kurrooa*	> 100	> 100	> 1.00	54.11 ± 7.42	> 100	> 1.84
*T. chebula*	3.24 ± 0.83	> 100	> 30.86	4.39 ± 1.31	> 100	> 22.77
*N. jatamansi*	75.32 ± 8.65	> 100	> 1.32	10.96 ± 2.13	> 100	> 9.12
Phikud Navakot formula	18.37 ± 2.60	> 100	> 5.44	15.64 ± 0.86	> 100	> 6.39
Artesunate	IC_50_ = 0.006 ± 0.01 μg/mL					
Doxorubicin	CC_50_ = 1.41 ± 0.09 μg/mL					

*Note:* Data are presented as the mean ± standard deviation (SD). IC_50_: 50% inhibition concentration; CC_50_: 50% cytotoxic concentration.

Abbreviation: ND, not determined.

**Table 5:** Identification of the chemical components in *T. chebula* gall extract using LC-QTOF-MS analysis.

No.	M/Z	Retention time (min)	Compound	Formula	Molecular weight (g/mol)
1	169.01	2.11	Gallic acid	C_7_H_6_O_5_	170.02
2	181.07	1.19	D-sorbitol	C_6_H_14_O_6_	182.07
3	179.03	7.12	Caffeic acid	C_9_H_8_O_4_	180.04
4	477.04	14.21	Chebulinic acid	C_41_H_32_O_27_	956.11
5	137.02	5.42	3,4-Dihydroxybenzaldehyde	C_7_H_6_O_3_	138.03
6	633.07	4.43	Pterocaryanin B	C_27_H_22_O_18_	634.08
7	331.06	1.44	4-Glucogallic acid	C_13_H_16_O_10_	332.07
8	953.08	11.31	Isoterchebin	C_41_H_30_O_27_	954.09
9	476.04	11.25	Isoterchebin	C_41_H_30_O_27_	954.09
10	285.04	22.15	Luteolin	C_15_H_10_O_6_	286.04
11	173.04	1.41	Shikimic acid	C_7_H_10_O_5_	174.05
12	781.05	1.90	Punicalin	C_34_H_22_O_22_	782.06
13	403.12	7.50	Oleoside 11-methyl ester	C_17_H_24_O_11_	404.13
14	1083.05	4.93	Punicalagin	C_48_H_28_O_30_	1084.06
15	541.02	2.31	Punicacortein D	C_48_H_28_O_30_	1084.06
16	393.11	4.74	2′-Oxoaloesol 7-glucoside	C_19_H_22_O_9_	394.12
17	285.06	3.94	Uralenneoside	C_12_H_14_O_8_	286.06
18	955.10	14.14	Chebulinic acid	C_41_H_32_O_27_	956.11
19	341.08	3.24	Glucocaffeic acid	C_15_H_18_O_9_	342.09
20	325.05	2.55	Fertaric acid	C_14_H_14_O_9_	326.06
21	300.99	12.51	Ellagic acid	C_14_H_6_O_8_	302.00
22	355.03	1.92	(+)-Chebulic acid	C_14_H_12_O_11_	356.03
23	651.08	7.25	Amlaic acid	C_27_H_24_O_19_	652.09
24	247.02	9.50	7-Deshydroxypyrogallin-4-carboxylic acid	C_12_H_8_O_6_	248.03
25	343.04	32.40	Aflatoxin GM1	C_17_H_12_O_8_	344.05
26	191.05	1.29	Quinic acid	C_7_H_12_O_6_	192.06
27	483.07	4.49	1,2′-Di-O-galloyl hamamelofuranose	C_20_H_20_O_14_	484.08
28	635.08	8.90	3-O-Galloyl hamamelitannin	C_27_H_24_O_18_	636.09
29	503.11	17.22	Rhamnocitrin 3-(6″-acetylglucoside)	C_24_H_24_O_12_	504.12
30	133.01	1.39	Malic acid	C_4_H_6_O_5_	134.02
31	483.07	4.21	1,2′-di-O-galloyl hamamelofuranose	C_20_H_20_O_14_	484.08
32	503.33	31.85	(3beta, 19alpha)-3,19,23,24-tetrahydroxy-12-oleanen-28-oic acid	C_30_H_48_O_6_	504.34
33	469.00	3.45	Sanguisorbic acid dilactone	C_21_H_10_O_13_	470.01
34	519.15	16.85	Chryso-obtusin glucoside	C_25_H_28_O_12_	520.15
35	355.03	1.60	(+)-Chebulic acid	C_14_H_12_O_11_	356.03
36	665.17	12.44	6″-O-Malonylnaringin	C_30_H_34_O_17_	666.17
37	541.02	3.59	Punicacortein D	C_48_H_28_O_30_	1084.06
38	827.22	9.09	Isorhamnetin 3-[2″-(4‴-acetylrhamnosyl)-gentiobioside]	C_36_H_44_O_22_	828.23
39	669.09	2.89	Myricetin 3,7-diglucuronide	C_27_H_26_O_20_	670.10
40	466.02	3.24	2-O-Galloylpunicalin	C_41_H_26_O_26_	934.07
41	785.08	8.53	Sanguiin H1	C_34_H_26_O_22_	786.09
42	503.15	19.63	Sergeolide	C_25_H_28_O_11_	504.16
43	300.99	12.81	Ellagic acid	C_14_H_6_O_8_	302.00
44	447.09	10.06	1,2,6,8-Tetrahydroxy-3-methylanthraquinone 2-O-b-D-glucoside	C_21_H_20_O_11_	448.10
45	633.07	7.71	Pterocaryanin B	C_27_H_22_O_18_	634.08
46	499.30	25.59	Ganolucidic Acid A	C_30_H_44_O_6_	500.31
47	695.40	29.30	Glucosyl passiflorate	C_37_H_60_O_12_	696.40
48	125.02	2.16	1,2,3-Trihydroxybenzene	C_6_H_6_O_3_	126.03
49	153.01	2.98	2,4-Dihydroxybenzoic acid	C_7_H_6_O_4_	154.02
50	331.06	1.74	4-Glucogallic acid	C_13_H_16_O_10_	332.07
51	311.04	1.94	Cis-caffeoyl tartaric acid	C_13_H_12_O_9_	312.04
52	275.01	9.62	5-(4-Acetoxybut-1-ynyl)-2,2′-bithiophene	C_14_H_12_O_2_S_2_	276.02
53	291.01	6.12	5-(3-Hydroxy-4-acetoxybut-1-ynyl)-2,2′-bithiophene	C_14_H_12_O_3_S_2_	292.02

**Table 6:** Assessment of the antimalarial potential of the *T. chebula* gall extract in *Plasmodium berghei*–infected mice.

Groups	Treatment dose (mg/kg)	% parasitemia	% suppression
PbA-infected	—	48.36 ± 3.34^b,c,d,e,f^	—
Artesunate	6	9.65 ± 1.52^a,c,d,e,f^	80.03 ± 2.82^c,d,e,f^
Chloroquine	25	0.66 ± 0.33^a,b,d,e,f^	98.62 ± 0.59^b,d,e,f^
*T. chebula*	200	29.55 ± 1.42^a,b,c,f^	38.88 ± 2.52^b,c,e,f^
	400	25.58 ± 1.21^a,b,c,f^	47.08 ± 2.23^b,c,d,f^
	600	20.02 ± 1.86^a,b,c,d,e^	60.61 ± 3.36^b,c,d,e^

*Note:* The antiplasmodial efficacy of the ethanolic extract of *T. chebula* gall at doses of 200, 400, and 600 mg/kg body weight was assessed using a 4-day suppression test in *Plasmodium berghei*–infected mice. The results are presented as mean ± standard deviation (SD), with five mice per treatment group (*n* = 5). Differences were considered statistically significant at *p* < 0.05.

^a^Compared to *Pb*A-infected.

^b^Compared to standard artesunate.

^c^Compared to standard chloroquine.

^d^Compared to 200 mg/kg dose of the *T. chebula* gall extract.

^e^Compared to 400 mg/kg dose of the *T. chebula* gall extract.

^f^Compared to 600 mg/kg dose of the *T. chebula* gall extract.

**Table 7:** Effect of the *T. chebula* gall extract on the body weights of infected mice in the 4-day suppressive test.

Groups	Mean body weight (g)		% changes
	Day 0	Day 4	
*Pb*A-infected	31.45 ± 1.37	31.22 ± 1.11	−0.68 ± 1.96^b,c^
Artesunate	36.16 ± 0.98	39.55 ± 1.48	9.37 ± 2.12^a,d,e,f^
Chloroquine	33.73 ± 1.99	36.05 ± 1.45	6.96 ± 2.24^a,d,e^
*T. chebula* 200 mg/kg	35.07 ± 1.79	35.01 ± 1.83	−0.15 ± 2.22^b,c^
*T. chebula* 400 mg/kg	33.48 ± 1.05	34.02 ± 1.21	1.63 ± 2.72^b,c^
*T. chebula* 600 mg/kg	33.55 ± 1.16	34.52 ± 0.75	2.93 ± 1.86^b^

*Note:* Data are expressed as mean ± standard deviation (SD) (*n* = 5/group), Day 0: day of infection, and Day 4: after completing treatment. Differences were considered significant at *p* < 0.05.

^a^Compared to *Pb*A-infected.

^b^Compared to standard artesunate.

^c^Compared to standard chloroquine.

^d^Compared to 200 mg/kg dose of the *T. chebula* gall extract.

^e^Compared to 400 mg/kg dose of the *T. chebula* gall extract.

^f^Compared to 600 mg/kg dose of the *T. chebula* gall extract.

**Table 8:** Assessment of the effects of aqueous extracts of *T. chebula* gall extracts on food and water intake during the acute toxicity evaluation at Week 1 and Week 2 post-treatment.

	Week 1	Week 2
Food consumption (g)		
Control group	21.42 ± 3.49	29.28 ± 1.74
*T. chebula* gall 2000 mg/kg	24.28 ± 4.16	28.57 ± 2.25
Water intake (mL)		
Control group	43.42 ± 6.75	43.28 ± 4.77
*T. chebula* gall 2000 mg/kg	45.00 ± 5.45	43.14 ± 1.95

*Note:* Data are expressed as the results are presented as mean ± standard deviation (SD) (*n* = 5/group). Differences were considered significant at *p* < 0.05.

**Table 9:** Assessment of the effects of aqueous extracts of *T. chebula* gall extracts on bodyweight changes during the acute toxicity evaluation at Week 1 and Week 2 post-treatment.

Group	Mean body weight		
	Week 1	Week 2	% change
Control group	33.98 ± 1.52	38.02 ± 2.01	11.88 ± 1.25
*T. chebula* gall 2000 mg/kg	33.65 ± 1.00	38.21 ± 1.46	13.52 ± 1.51

*Note:* Data are expressed as mean ± standard deviation (SD) (*n* = 5/group). Differences were considered significant at *p* < 0.05.

**Table 10:** Effect of the *T. chebula* gall extract on liver and kidney functions in acute toxicity evaluation.

Biochemical parameters	Control group	2000 mg/kg
Live parameters		
AST (U/L)	48.56 ± 6.51	56.48 ± 8.24
ALP (U/L)	166.40 ± 21.14	161.80 ± 15.02
ALT (U/L)	26.54 ± 3.75	27.30 ± 1.34
Kidney parameters		
BUN (mg/dL)	19.64 ± 0.67^b^	16.88 ± 0.47^a^
Creatinine (mg/dL)	0.07 ± 0.01	0.07 ± 0.01

*Note:* Data are expressed as mean ± standard deviation (SD) (*n* = 5/group). Differences were considered significant at *p* < 0.05.

^a^Compared to normal mice.

^b^Compared to 2000 mg/kg crude extract.

## Data Availability

The data that support the findings of this study are available from the corresponding author upon reasonable request.
